# Food Consumption and Nutrient Intake by Children Aged 10 to 48 Months Attending Day Care in The Netherlands

**DOI:** 10.3390/nu8070428

**Published:** 2016-07-14

**Authors:** R. Alexandra Goldbohm, Carina M. Rubingh, Caren I. Lanting, Koen F. M. Joosten

**Affiliations:** 1 Netherlands Organisation for Applied Scientific Research TNO, Schipholweg 77–89, Leiden 2316 ZL, The Netherlands; 2 Netherlands Organisation for Applied Scientific Research TNO, Utrechtseweg 48, Zeist 3700 AJ, The Netherlands; carina.dejong@tno.nl; 3 Erasmus University Medical Centre, Erasmus MC-Sophia Children’s Hospital, Dr. Molewaterplein 60, Rotterdam 3015 GJ, The Netherlands; k.joosten@erasmusmc.nl

**Keywords:** young children, nutrition, dietary habits, childcare

## Abstract

The diet of young children is an important determinant of long-term health effects, such as overweight and obesity. We analyzed two-day food consumption records from 1526 young children (10–48 months old) attending 199 daycare centers across The Netherlands. Data were observed and recorded in diaries by caregivers at the day nursery and by parents at home on days that the children attended the daycare center. According to national and European reference values, the children had an adequate nutrient intake with exception of low intakes of total fat, *n*-3 fatty acids from fish and possibly iron. Intakes of energy and protein were substantially higher than recommended and part of the population exceeded the tolerable upper intake levels for sodium, zinc and retinol. Consumption of fruit, fats, fish, and fluids was substantially less than recommended. The children used mostly (semi-)skimmed milk products and non-refined bread and cereals, as recommended. Two thirds of the consumed beverages, however, contained sugar and contributed substantially to energy intake. In young children, low intakes of *n*-3 fatty acids and iron are a potential matter of concern, as are the high intakes of energy, protein, sugared beverages, and milk, since these may increase the risk of becoming overweight.

## 1. Introduction

Proper nutrition of young children promotes optimal growth and development. The right amount of energy and nutrients also reduces the risk for developing overweight and obesity, dental caries, and gastrointestinal problems like constipation and diarrhea. In addition, there is some evidence that diet quality and especially breakfast consumption is related to cognitive functioning and academic performance of children [1,2]. Unfavorable dietary habits may have long-term implications, especially since childhood overweight tends to track into adulthood [3] and overweight and obesity are major risk factors for cardiovascular disease, type 2 diabetes mellitus, and cancer [4]. Early childhood is a critical period for prevention of diet-related disease later in life; dietary habits tend to be established at an early age and are maintained throughout later life [5].

In The Netherlands, about 40% of children until four years of age attend childcare [6]. Childcare providers are in a unique position to educate parents about healthy eating, to encourage children to eat healthy, and to provide a healthy environment for children to eat, grow and develop. In the current study, food consumption was registered in a large population of young children (10–48 months old) attending day care across The Netherlands. Intakes of energy, macro- and micronutrients and on consumption of a complete set of food groups (e.g., bread, milk products, sweetened beverages) were established and compared with current recommendations. Some of the results have been published previously [7,8]; however, these were based on a smaller sample and only comprised a small selection of macronutrients and a few food groups.

## 2. Materials and Methods

### 2.1. Study Population

Details on the recruitment methods were previously published [7]. In brief, 199 childcare centers located across the Netherlands, were recruited between 2011 and 2014 to participate in the study, which was originally offered to all childcare centers in The Netherlands by Nutricia Nederland BV, Zoetermeer, The Netherlands, as a service to support their nutrition policy. Children between the age of 10 and 48 months who were attending the nursery for at least two days per week could participate in the study. The manager of the childcare center invited the children’s parents to participate in the study and obtained written and signed informed consent from them. The study has been conducted in accordance with the Declaration of Helsinki and complied with national and EU data protection laws (Directive 95/46/EC). According to Dutch law, approval by a medical-ethical committee is exempted for this type of observational research.

### 2.2. Dietary Intake Assessment

Details on the assessment methods were previously published [7,8]. In brief, at the childcare center, food and beverage consumption was recorded on a structured poster by the carers for all children participating in the study during two days. Dutch children attending a childcare center usually eat breakfast and the main (hot) meal at home and lunch and a morning and afternoon snack at the childcare center. Few childcare centers serve a hot meal during lunchtime or in the evening. For the food and beverages consumed at home, a parent completed a structured diary at the same two days. Berdien van Wezel Dieticians (The Hague, The Netherlands), were responsible for data collection. Trained dieticians instructed carers and parents, supervised the recording and were responsible for coding and computer entry of the consumption data using the FoodFigures system [9].

### 2.3. Data Processing

A raw data set containing, for each child, records with food codes and a quantity (g or mL), eating occasion (e.g., breakfast), day and location (home or childcare center) per food code was exported from the FoodFigures system. Data checking and calculation of nutrient intake and food group consumption was performed using a software application in SAS [10,11] specifically developed for processing of dietary data [12]. The Dutch food composition database (NEVO), edition 2013, was used for calculation of nutrient intake [13]. In this edition of the NEVO database, dietary fiber is considered an energy-contributing nutrient (2 kcal/g fiber). A few food codes were derived from the NEVO database of 2011 or from an additional food composition database. Data were checked for inconsistencies and completeness of the records, i.e., two days, both at the childcare center and at home, should be present for each child. Potential outliers at the high and the low end of the quantity of consumed foods were listed and checked in the original diaries and posters. After correction of the database, a final check on potential outliers of the calculated nutrient intake was performed.

### 2.4. Data Analysis

Mean daily energy and nutrient intake was calculated for all children and for each age group (i.e., 10 and 11 months old and 1, 2 and 3 years old, which included children ≥12–<24 months, ≥24–<36 months, and ≥36–<48 months, respectively). As (energy-adjusted) nutrient intake differed very little between boys and girls, their results are not separately presented. We used the “Statistical Program to Assess Dietary Exposure” (SPADE) to transform the nutrient intake from the two days into a theoretical “usual” intake distribution of the population [14]. For all nutrients, we used the SPADE model for daily intakes, except for *n*-3 fish fatty acids and folic acid for which the module for episodical intakes was used and docosahexaenoic acid, for which no model was found that fitted the required criteria. We followed the European Food Safety Authority’s (EFSA) guidance [15] to compare the usual intake distribution with dietary reference values (specifically, Adequate Intake and Tolerable Upper Intake Levels (TUIL)) as established by the Health Council of The Netherlands (HCN) [16,17,18] and, if relevant, more recent ones from the EFSA [19]. For this comparison, we assumed that the usual intake based on the two weekdays that the children attended the childcare center is representative for their true usual intake.

Furthermore, individual foods were divided into food groups to calculate the mean daily consumption of each food group. We defined several food group classifications. The first, a classic one, was based on type of food (e.g., meat, meat products, and poultry; bread; fruit). We used a second one, based on the food based dietary guidelines (“Schijf van Vijf”) published by The Netherlands Nutrition Centre (NNC) [20], to compare mean daily consumption of the relevant food groups with the recommendations that apply to children from 1 to 3 years old. Finally, we subdivided the foods grouped according to the NNC classification in preferable or neutral foods and foods that should be eaten by exception only [21]. Preferable foods contribute positively to a diet preventive for chronic diseases, whereas “by exception” foods contribute negatively to such a diet. Classification of foods is based on their content of saturated fat, trans fat, sodium, dietary fiber and added sugar. For example, full-fat milk products (as opposed to skimmed milk) are classified as by-exception food, just as milk products and beverages with added sugar. The contribution of these food groups to the intake of selected nutrients was also calculated. Furthermore, we simulated the effect of replacing liquid cow’s milk by formula among exclusive cow’s milk users on usual iron and protein intake. In this simulation, liquid cow’s milk was replaced by young children’s formula (with a nutrient composition according to Nutrilon, Zoetermeer, The Netherlands, a brand from the Nutricia company).

## 3. Results

### 3.1. Study Population

Table 1 presents the characteristics of the study population (*n* = 1526). Body weight and height are not presented, as they were not considered sufficiently accurate and comprised a high proportion of missing values.

Between 19 and 67 (mean 41) food records were available per child. A food record comprises the consumption of a given quantity of a given food or beverage at a given occasion (e.g., breakfast). 14 children were breastfed (11 of whom were 12 months or younger).

### 3.2. Nutrient Intake

Table 2 displays the average daily intake of macronutrients (mean and standard deviation), both in absolute amount as in energy density. Boys had a higher mean energy intake than girls (5483 kJ versus 5271 kJ). All gender differences in intake of macronutrients were attributable to the higher energy intake by boys. However, the intake relative to energy was very similar for boys and girls (see supplementary materials Table S1). The absolute intakes of micronutrients (Table 2) were also somewhat higher for boys than for girls (see supplementary materials Table S1). According to their parents, 92% of the children had received a vitamin D supplement on at least one of the two study days.

Comparing the transformed usual intake distribution with dietary reference values the following results were found. The intake of most of the assessed macro- and micronutrients was found to be adequate (see supplementary materials Table S2). Exceptions were encountered for:

- Energy: Mean daily intake, both for boys (5.5 MJ) and girls (5.3 MJ), was higher than the requirement established by the HCN (5 MJ for boys, 4.5 MJ for girls between 1 and 3 years old [16] and much higher than the intake considered adequate according to EFSA (3.3–4.9 MJ for boys, 3.0–4.6 MJ for girls [19]).
- Protein: Median usual intake (45 g, 14 energy percent) was approximately three times higher than the Adequate Intake of 5 energy percent [16] or the Population Reference Intake of 11–13 g [19]. Among the children below 1 year, 26% exceeded the TUIL of 15 energy percent for this age. If, among the children that used exclusively cow’s milk, liquid cow’s milk would be substituted by formula, median usual protein intake would decrease from 14.2 to 12.3 energy percent.
- Fat: Intake was relatively low (29 energy percent). The mean usual intake was within the recommended range (25–40 energy percent) according the HCN recommendation [16], although 11% of the population had an intake below 25 energy percent. The mean intake was lower than the more recent EFSA recommendation (35–40 energy percent for children under 4 years [19]). Less than 5% of the children achieved a usual fat intake of 35 energy percent. No children below the age of 1 year achieved a usual fat intake of 40 energy percent, as recommended by the HCN and EFSA.
- Dietary fiber: With a mean of 12.7 g or 2.3 g/MJ, the usual intake of the children of 1 year and older was below the intake considered as adequate by the HCN (2.8 g/MJ [17]) and not achieved by 92% of them. However, new reference intakes for young children established by EFSA are lower (10 g [19]). These were achieved by the large majority of the children.
- *n*-3 fatty acids from fish, i.e., eicosapentaenoic acid (EPA) and docosahexaenoic acid (DHA): the mean daily intake of DHA by children below 2 years and of EPA + DHA by children above 2 years was far below the intake considered adequate by the HCN or EFSA.
- Iron: The median usual iron intake of 6.0 mg was lower than the Adequate Intake of 8 mg/day [18] and also lower than the Population Reference Intake of 7 mg/day established by EFSA [22]. This implies that iron intake was possibly inadequate. Children receiving formula, most of them in combination with cow’s milk, had a median usual intake of 6.8 mg iron, whereas exclusive cow’s milk users had a median usual intake of 5.6 mg. If, in the latter group, liquid cow’s milk products would be replaced by formula, median usual iron intake would increase to 8.5 mg.
- Sodium: The percentage of children with a usual sodium intake of more than 1200 mg, i.e., the upper limit established by the NNC for young children above 1 year, increased with age from 47% to 81%, whereas the usual intake of all children below 1 year exceeded the upper limit of 400 mg [23].
- Zinc: The median usual intake of 5.9 mg was adequate. However, 17% of the children exceeded the TUIL of 7 mg/day.
- Retinol: 30% of the children exceeded the TUIL of 800 mg retinol. The percentage increased from 27% in the youngest age group to 33% in the oldest age group. Liverwurst spread, popular among young children, appeared to be responsible for the excess intake.

### 3.3. Food Consumption

Almost all children (98%) had eaten a breakfast on both days, contributing to 20% of the daily energy intake. Only 12 out of 1526 (<1%) children did not have breakfast on any of the days.

Table 3 shows the mean daily consumption of food groups, classified according to type of food, across all age groups. Consumption of potatoes, vegetables, fruits, cereals, and cheese was similar across all age groups. In contrast, consumption of bread and, in accordance, fat spreads doubled over age, just as cakes and biscuits, and meat. Consumption of milk products increased slightly over age, with exception of the much lower consumption (176 g) by children under 1 year. However, this was compensated by the high consumption (411 mL) of follow-on formulae among the youngest age group, 87% of which used formula. Mean consumption of formulae (follow-on or young-child formulae) decreased from 111 mL among the 1-years old (41% users) to 15 mL among the 3-years old (7% users). Consumption of mixed dishes, including baby and young-child meals in jars, also substantially decreased over age. Mean fish consumption was rather low, and did not change with age. We calculated from the food records that the children ate fish once per 10 days on average.

### 3.4. Consumption According to Food-Based Dietary Guidelines

**Figure 1:**
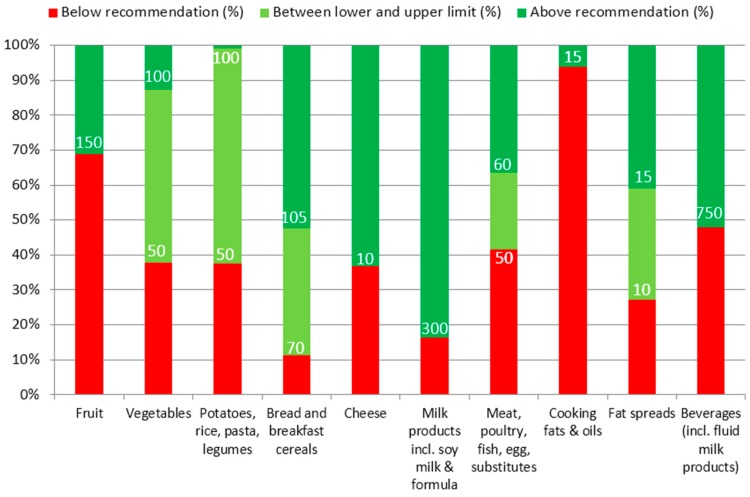
Percentage of children from 1 year old with a daily consumption (g) below, above or in within the range of the recommendation.

Figure 1 displays the consumption of food groups by children from 1 year, classified according to the food-based dietary guidelines. Figure 1 shows for each defined food group the percentage of children with a consumption quantity (g or mL) below and above the recommended daily consumption. The majority of children did not comply with the recommendations for fruit and cooking fats. Around 40% of the children did not comply with the recommendations for vegetables, potatoes (including rice and pasta), cheese, meat (including poultry, fish and eggs) and beverages (including liquid milk products). Children who ate a hot meal at the childcare center ate more vegetables: Only 30% of them did not comply with the recommendation. A large majority of children complied with the recommendations for bread (including breakfast cereals), fat spreads and milk products.

The food groups were also subdivided into preferable or neutral foods and foods that should be eaten by exception only. Table 4 shows the mean consumption of each food group. More than half of the foods from the potatoes group were eaten as by-exception type. This was due to the consumption of white rice and refined pasta (instead of brown rice and wholemeal pasta). In contrast, bread and breakfast cereals were almost always eaten as wholemeal or “brown” type. Cheese was mostly eaten as full-fat type and therefore classified as by-exception type. Milk products were mostly (79%) consumed as preferable or neutral type, i.e., (semi-)skimmed, non-sweetened milk. Two thirds of the meat group was eaten as by-exception type, mostly because of the saturated fat content of the meat or the salt content of processed meats. Fats and oils, in contrast, were predominantly of the preferable (less-saturated fat) type. Sixty-two percent of the beverages were drunk as by-exception type, mostly because they contained sugars. This percentage is even higher as part of the water (in the preferable group) was used to mix it with fruit juice concentrate or syrup. Thirty-seven percent of the total foods (in g) consumed by the children were by-exception foods (35% at home and 40% at the childcare center). The higher consumption of by-exception foods at childcare centers was mainly attributable to by-exception beverages (see supplementary materials Figure S1).

Table 4 also lists the contribution of the food groups to mean intake of selected nutrients. Bread, milk products and sweetened beverages were important contributors to energy intake. Milk products contributed most to protein intake, followed by bread and the meat group. Sweetened beverages were by far the largest contributors to intake of sugars, at a distance followed by milk products (contributes mainly lactose), fruit, and sugar and sweet spreads. Bread and potatoes were the main contributors to polysaccharides. Milk products, meats, and fats and oils contributed mostly to intake of fat and saturated fatty acids. However, almost half of the intake of polyunsaturated fatty acids originated from fats and oils. Dietary fiber came mainly from bread and breakfast cereals, followed by vegetables and fruit. Bread was also responsible for one third of the daily sodium intake (which excluded salt added at cooking and at the table), followed by processed meats, cheese and milk products. Fruit, followed at a distance by sweetened beverages (including fruit syrups and juices) and vegetables and milk, contributed most to vitamin C intake.

## 4. Discussion

This study showed that Dutch children aged 10 to 48 months, at the days they attended childcare, appeared to have an adequate nutrient intake according to national and European reference values with exception of intakes of total fat, *n*-3 fatty acids from fish and possibly iron which were all lower than the reference values. Intakes of energy and protein were substantially higher than recommended and part of the population exceeded the Tolerable Upper Intake Levels for sodium, zinc and retinol. Consumption of fruit and fats was substantially less than recommended according to food-based dietary guidelines. So were, to a lesser extent, components of the hot meal (vegetables, potatoes/rice/pasta, and meat/fish/poultry/eggs) and fluids. The children consumed milk products and bread mostly as recommended with respect to type (i.e., low fat and high fiber types, respectively), but milk consumption was on average higher than recommended. A notably high consumption of sugar-containing beverages contributed substantially to energy intake. Almost all children ate breakfast and received vitamin D supplements at the recorded days.

Results presented here are from a large dataset, which covers a very large variety of childcare centers, located across the country. Some limitations should be mentioned. Firstly, the authors were not involved in the design of the data collection methods, nor in the execution of the data collection. It was therefore difficult to evaluate the quality of the data. However, based on the documentation provided and the performed data checks, we assessed the data to be of sufficient quality to merit publication. Missing data on gender, age, and body weight and height reduced the usefulness of the data. However, comparing our results with those of the Dutch National Food Consumption Survey conducted among young children in 2005/2006 in the age categories of 2- and 3-years old overlapping between the studies, we observed that mean energy intake was very similar between the surveys (5632 kJ/day versus 5645 kJ/day, respectively). The Dutch National Food Consumption Survey concluded, based on comparison of energy intake and energy requirement, that underreporting was not an issue in their survey [24]. We conclude therefore that substantial underreporting is unlikely in our study either. Secondly, there are considerations with respect to the study population and design. During the period of data collection (2011–2014) approximately 40% of Dutch children under the age of 4 attended a childcare center on one or more days per week [6]. Although formal childcare in the Netherlands is partly subsidized depending on family income, parents (in particular mothers) of children attending a childcare center had on average a higher family income, were more educated and worked more hours per week than parents of other children of the same age [25]. The study population was therefore not representative of the total Dutch population of 10 to 48 months old. In addition, response rates were not formally monitored. Furthermore, food consumption was only recorded on days that the children attended the childcare center. Although we assumed—to compare the children’s usual nutrient intake distribution with dietary reference values—that the recorded days were representative of a child’s diet, this can be questioned, considering the structure of the day, the food provider, and possible peer pressure. Nevertheless, the data are valuable in their own right. They were mostly observed and recorded directly by the food providers, both at home and at the childcare center. The latter is unusual in food consumption surveys.

As concluded from other studies and countries, reviewed by EFSA [19], intake of *n*-3 fatty acids, in particular DHA and EPA, and iron and vitamin D is low among the Dutch young children. The low dietary intake of vitamin D was largely remedied through the high proportion of children (92%) that received a vitamin D supplement. Although some practitioners doubt whether such a high percentage of supplement users is real, communication about the recommendation has improved since 2012. Intake of *n*-3 fatty acids was much lower than recommended. Mean DHA intake by children younger than 2 years is only one fifth of the intake considered as adequate (100 mg/day). Children receiving formula, fortified with DHA, more or less double their DHA intake. If the children of 2 to 4 years old would eat 50 g lean fish and 50 g fatty fish such as salmon per week, as recommended by the NNC, they would easily achieve an adequate intake of DHA and EPA combined. Whether the low *n*-3 fatty acid intake has health consequences is uncertain. As for iron, there is evidence that iron deficit at young age may interfere with cognitive development [26,27]. However, while the usual iron intake of all children in this survey is above the Average Requirement of 3 mg/day for 1–3 years old established by the US Institute of Medicine [28], 25% of the children from 1 year do not achieve the Average Requirement of 5 mg/day recently proposed by EFSA [22]. Recent research in a well-defined, healthy population of 400 young children in The Netherlands has shown that iron deficiency and iron deficiency anemia was detected in 18.8% and 8.5% of the children, respectively, with a lower iron deficiency prevalence among children receiving formula and a higher prevalence among children receiving a large amount (more than 400 mL/day) of cow’s milk after adjustment for age [29]. However, iron intake was not assessed in this study.

The high intake of energy and protein among young children is also a universal observation across Europe [19]. They both increase the risk for overweight [30,31]. In particular the 1-year-old are at risk: they use a lot of milk, mostly cow’s milk and also formula, and have the highest protein intake. Although no reference value for sugars has been established by the HCN or by EFSA, increasing evidence shows that in particular sugar-sweetened beverages increase the risk of overweight [32]. We also found that usual intake exceeded the Tolerable Upper Intake Level for zinc (17%) and retinol (30%). As the Adequate Intake and TUIL for zinc are very close, such an excess is inevitable and is unlikely to be harmful. The TUIL for retinol intake among young children is based on the relatively low TUIL for pregnant women owing to its teratogenic effects and adjusted to children. Some excess intake is therefore not likely to be harmful for young children. Also, vitamin A intoxication has not been reported in young children in The Netherlands. Excess retinol intake can nevertheless be avoided if childcare centers and parents restrict the consumption of liverwurst (spread) by their young children, in accordance with the NNC guideline. Dietary sodium intake—70% of the children exceeded the TUIL set by the NNC—is difficult to reduce without endangering a balanced diet. Even though the Dutch bread sector made a major effort to reduce the salt content of bread, bread is a main contributor to young children’s sodium intake. According to EFSA, however, sodium intake is not a matter of concern [19].

The results of this survey are in particular informative to nutrition policy and education, both for childcare organizations as for parents and youth health care providers. They demonstrate that the young children’s food consumption pattern could be substantially improved by a few changes, such as a replacement of sugared beverages by water and some reduction of milk products. For example, milk-based desserts could be replaced by fruit. Childcare centers that serve a hot meal are able to increase the children’s daily vegetable consumption. All in all, these changes would result in lower energy and protein intake and higher vegetable and fruit consumption, which may in turn lower the risk of overweight. Reduction of a high milk consumption should be able to enhance iron absorption from the diet, as calcium hampers iron absorption. Stimulating fish consumption, both at home and at day care, is a feasible way to increase intake of *n*-3 fatty acids. Only if, in specific situations, a recommended diet is difficult to achieve, replacement of cow’s milk by formula may help to meet some of the dietary recommendations.

## 5. Conclusions

Dutch 10 to 48-month-old had, at least on the days they attended childcare, mostly an adequate nutrient intake. The intake of *n*-3 fatty acids and of iron however was low, which is in line with European findings. This seems a matter of concern in young children in this survey and most likely in all young children in the Netherlands, although more research is needed on their potential health effects. The high intakes of energy and protein in this population are also a matter of concern as they may increase the risk of becoming overweight.

Almost all children ate breakfast and received vitamin D supplements. They used mostly (semi-) skimmed types of milk products and non-refined bread and cereals, as recommended. However, their relatively high milk consumption and very high consumption of sugared beverages are undesirable and the latter should be replaced mostly by water. The relative low consumption of fruit, vegetables and fish, on the other hand, should be increased.

## Figures and Tables

**Table 1:** Characteristics of the study population.

		*n*	%
Gender	Boy	759	49.7
	Girl	683	44.8
	Missing	84	5.5
Age	10–11 months old	31	2.0
	1 year old	411	26.9
	2 years old	497	32.6
	3 years old	410	26.9
	Missing	177	11.6
Zip-code based socioeconomic status of childcare centers	Low	381	25.0
	Medium	848	55.6
	High	218	14.3
	Missing	79	5.2
Number of days per week that the child attended childcare center	2 days	939	61.5
	3 days	348	22.8
	4 days	68	4.5
	5 days	16	1.1
	Missing	155	10.2
Hot meal at childcare center	Yes	315	20.6
	No	1189	77.9
	Missing	22	1.4

**Table 2:** Mean daily nutrient intake by children attending childcare by age (mean of 2 days).

		Total Group (*n* = 1526) ^1^		10–11 Months (*n* = 31)		1 Year (*n* = 411)		2 Years (*n* = 497)		3 Years (*n* = 410)	
Nutrient	Unit	Mean	SD	Mean	SD	Mean	SD	Mean	SD	Mean	SD
Energy	kJ	5383	958	4241	795	4954	822	5424	872	5841	923
Protein	g	45	9	32	8	43	9	45	8	48	10
	en%	14.1	2.2	12.6	2.2	14.6	2.4	13.9	2.0	13.9	2.1
Carbohydrates	g	174	34	136	27	159	30	177	32	189	33
	en%	54.4	5.1	54.2	5.9	54	5.3	54.8	5.0	54.5	4.8
Mono- and disaccharides	g	100	25	79	18	91	24	102	24	108	26
	en%	31.2	5.7	31.4	5.5	30.7	6.1	31.7	5.3	31.1	5.7
Polysaccharides	g	75	16	58	15	69	14	75	15	81	16
	en%	23.4	3.4	23.1	3.6	23.6	3.6	23.3	3.1	23.4	3.3
Fat	g	42	11	34	10	38	10	42	10	45	11
	en%	29.1	4.6	30.4	5.5	28.7	4.8	28.9	4.5	29.3	4.3
Saturated fatty acids	g	14.8	3.8	12.6	4.7	13.5	3.4	14.8	3.7	16.1	3.8
	en%	10.4	1.8	11.2	2.8	10.3	1.9	10.3	1.8	10.4	1.7
Monounsaturated fatty acids	g	14.4	4.5	11.7	4.3	12.9	4.2	14.4	4.5	15.8	4.4
	en%	10.0	2.3	10.4	3.0	9.8	2.4	9.9	2.3	10.2	2.2
Polyunsaturated fatty acids (PUFA)	g	8.6	3.2	6.7	2.3	8.0	3.1	8.6	3.0	9.4	3.4
	en%	6.0	1.8	5.9	1.5	6.1	1.9	5.9	1.7	6.0	1.7
*n*-3 PUFA	g	1.0	0.4	0.9	0.3	1.0	0.3	1.0	0.4	1.1	0.4
	en%	0.7	0.2	0.8	0.2	0.7	0.2	0.7	0.2	0.7	0.2
ALA (C18:3 (*n*-3) *cis*)	g	1.0	0.3	0.8	0.3	0.9	0.3	1.0	0.3	1.0	0.4
	en%	0.7	0.2	0.7	0.2	0.7	0.2	0.7	0.2	0.7	0.2
EPA (C20:5 (*n*-3) *cis*)	g	0.01	0.03	0.02	0.03	0.02	0.03	0.01	0.02	0.01	0.03
	en%	0	0	0	0	0	0	0	0	0	0
DHA (C22:6 (*n*-3) *cis*)	g	0.02	0.04	0.02	0.04	0.02	0.05	0.02	0.04	0.02	0.04
	en%	0	0	0	0	0	0	0	0	0	0
*n*-6 PUFA	g	7.3	2.8	5.4	2.0	6.8	2.7	7.3	2.6	8.0	3.0
	en%	5.1	1.6	4.8	1.4	5.1	1.7	5.0	1.5	5.1	1.5
Linoleic acid (C18:2 (*n*-6) *cis*)	g	7.3	2.8	5.4	2.0	6.7	2.7	7.2	2.6	7.9	3.0
	en%	5.0	1.6	4.8	1.4	5.1	1.7	5.0	1.5	5.1	1.5
Trans-unsaturated fatty acids	g	0.4	0.2	0.3	0.2	0.4	0.2	0.4	0.2	0.5	0.2
	en%	0.3	0.1	0.2	0.1	0.3	0.1	0.3	0.1	0.3	0.1
Cholesterol	mg	76.1	38.0	45.9	45.9	69.2	37.2	77.4	38.6	84.4	38.2
Dietary fiber	g	12.6	2.7	12.4	2.6	12.4	2.7	12.5	2.6	13.1	2.7
	g/MJ	2.4	0.4	3.0	0.5	2.5	0.5	2.3	0.4	2.3	0.4
Water	g	1045	248	885	219	988	240	1047	231	1133	245
Sodium	mg	1273	359	790	266	1117	301	1317	340	1420	341
Potassium	mg	1923	405	1435	420	1864	391	1934	377	2010	402
Phosphorus	mg	943	199	715	190	907	189	941	186	997	206
Magnesium	mg	181	38	134	29	172	36	181	34	194	40
Iron	mg	6.3	2.0	8.4	2.5	6.5	2.4	6.0	1.7	6.1	1.7
Calcium	mg	735	186	684	200	734	177	720	177	745	202
Copper	mg	0.61	0.15	0.59	0.13	0.58	0.14	0.60	0.14	0.66	0.15
Selenium	μg	22	6	21	6	21	6	22	6	24	6
Zinc	mg	6.0	1.4	6.0	1.4	6.0	1.4	5.9	1.4	6.1	1.5
Iodine	μg	120	30	118	22	117	31	119	30	124	31
Vitamin A (RAE)	μg	846	568	753	448	746	483	834	568	902	626
Retinol	μg	731	560	648	434	636	479	712	558	787	618
Thiamin (Vitamin B_1_)	mg	0.58	0.17	0.60	0.20	0.58	0.17	0.57	0.17	0.57	0.17
Riboflavin (Vitamin B_2_)	mg	1.13	0.29	0.97	0.24	1.09	0.27	1.12	0.29	1.17	0.31
Niacin	mg	7.7	2.5	6.4	2.6	7.4	2.4	7.7	2.5	8.1	2.6
Vitamin B6	mg	0.80	0.27	0.69	0.28	0.79	0.25	0.81	0.28	0.82	0.28
Folate (DFE)	μg	156	47	177	49	162	47	154	50	154	46
Vitamin B_12_	μg	2.95	1.06	1.95	0.79	2.73	0.90	2.94	1.00	3.18	1.19
Vitamin C	mg	93	35	105	29	94	35	92	35	92	35
Vitamin D	μg	3.7	2.8	8.4	3.7	4.7	3.5	3.2	2.1	2.8	1.8
Vitamin E	mg	6.8	2.8	8.0	2.9	6.8	3.0	6.6	2.7	7.0	2.7

^1^ The total number of children does not equal the sum of those in the age groups, due to missing data on age. en%: energy percent, i.e., the energy that the nutrient contributes to the total energy intake of a person, expressed as percentage; ALA: alpha-linolenic acid; EPA: eicosapentaenoic acid; DHA: docosahexaenoic acid; RAE: retinol activity equivalents; DFE: dietary folate equivalents; SD: standard deviation

**Table 3:** Mean daily consumption of food groups (g) by children attending childcare by age (mean of 2 days).

	Total Group (*n* = 1526) ^1^		10–11 Months (*n* = 31)		1 Year (*n* = 411)		2 Years (*n* = 497)		3 Years (*n* = 410)	
**Food Group**	**Mean**	**SD**	**Mean**	**SD**	**Mean**	**SD**	**Mean**	**SD**	**Mean**	**SD**
Potatoes	29	27	25	25	31	28	28	27	28	26
Vegetables	58	39	48	45	60	41	58	39	61	38
Fruit	128	59	129	66	127	58	129	59	130	61
Legumes	1	7	1	4	2	9	1	6	1	6
Bread	100	35	57	29	86	33	103	33	114	33
Cereals (including rice and pasta)	29	29	21	26	30	30	28	28	30	28
Cakes and biscuits	13	12	6	8	11	10	14	12	16	13
Nuts, seeds, savory snacks	1	5	0	0	1	3	1	5	2	6
Milk and milk products	358	158	176	217	333	162	363	143	383	147
Follow-on and young children’s formula	63	135	411	226	111	162	35	87	15	61
Cheese	14	11	14	10	15	11	14	11	15	12
Eggs	3	8	1	4	2	7	3	8	3	8
Fish	5	12	2	7	5	12	5	12	6	13
Meat, meat products, poultry	44	25	24	23	40	24	44	22	49	26
Soy and vegetarian products	8	49	0	2	8	54	8	47	10	55
Savory spreads	4	6	0	0	3	6	4	6	4	7
Mixed dishes	20	45	52	78	28	53	15	39	14	35
Soups	10	33	0	1	6	23	12	37	13	39
Sugar, confectionary, sweet spreads	18	13	6	7	13	10	20	12	23	14
Fat spreads, oils, savory sauces	24	11	11	8	20	10	24	11	27	12
Non-alcoholic beverages (excluding milk products)	427	222	182	145	367	204	446	209	508	220

^1^ The total number of children does not equal the sum of those in the age groups, due to missing data on age; SD: standard deviation.

**Table 4:** Consumption of food groups ^1^ and their contribution to intake of selected nutrients among children 1–3 years old.

	Mean	Energy	Protein	MDS	PS	Fat	SFA	PUFA	Water	Dietary Fiber	Sodium	Vitamin C
**Basic Foods**	**g/day**	**%**	**%**	**%**	**%**	**%**	**%**	**%**	**%**	**%**	**%**	**%**
Fruit	123	6	2	13	3	1	1	2	10	16	0	42
Vegetables	58	1	2	1	1	1	0	1	5	10	1	11
Potatoes, rice, pasta, legumes	23	2	1	0	5	0	0	1	2	4	0	2
Potatoes, rice, pasta, legumes (exception)	30	3	3	0	10	2	1	2	2	3	0	1
Bread and breakfast cereals	98	19	22	3	54	4	3	7	3	42	34	2
Cheese	1	0	0	0	0	0	1	0	0	0	1	0
Cheese (exception)	13	3	5	0	0	8	13	1	1	0	10	0
Milk products including soy milk and formula	333	13	23	16	1	13	21	4	26	4	10	11
Milk products incl. soy milk (exception)	90	6	7	9	2	5	8	2	7	2	4	1
Meat, poultry, fish, egg, substitutes	18	2	9	0	0	2	2	2	1	0	4	0
Meat, poultry, fish, egg, substitutes (exception)	35	8	15	0	2	18	18	12	2	1	13	6
Fats, oils	18	8	0	0	0	26	15	47	1	0	2	0
Fats, oils (exception)	1	0	0	0	0	1	1	1	0	0	0	0
Beverages, excluding milk products	165	0	0	0	0	0	0		16	0	0	1
Beverages, excluding milk products (exception)	267	12	1	39	0	0	0	0	22	3	4	21
Mixed dishes	12	1	1	0	1	1	1	2	1	2	1	0
Mixed dishes (exception)	7	1	1	0	2	1	2	1	0	1	2	0
**Non-Basic Foods**												
Sugar, sweet spreads	18	5	1	12	1	4	4	2	0	3	0	0
Cakes and biscuits	13	4	2	4	7	3	4	2	0	3	3	0
Confectionary	3	1	0	1	0	1	1	0	0	0	0	0
Nuts, seeds, savory snacks	1	0	0	0	1	1	1	1	0	0	1	0
Sauces	5	1	0	0	0	2	1	4	0	0	2	0
Savory spreads	4	2	2	0	0	5	2	5	0	2	1	0
Soups	9	0	0	0	0	0	0	1	1	0	2	1
Miscellaneous ^2^	14	3	2	1	8	1	1	1	1	4	4	0

^1^ Division in food groups according to food choice guidelines by the Netherlands Nutrition Center [21]; ^2^ The sum of all not listed food groups that were consumed in small quantities; MDS: mono- and disaccharides; PS: polysaccharides; SFA: saturated fatty acids; PUFA: polyunsaturated fatty acids.

## Supplementary Materials

The following are available online at http://www.mdpi.com/2072-6643/8/7/428/s1, Figure S1: Consumption of foods by 1–3 years old by location, Table S1: Mean daily nutrient intake by children 10–48 months old attending childcare by gender (mean of 2 days), Table S2: Distribution of usual nutrient intake as calculated with SPADE model for daily intakes and comparison of the distribution with dietary reference values.

## References

[1] Hoyland A., Dye L., Lawton C.L. (2009). A systematic review of the effect of breakfast on the cognitive performance of children and adolescents. Nutr. Res. Rev..

[2] Florence M.D., Asbridge M., Veugelers P.J. (2008). Diet quality and academic performance. J. Sch. Health.

[3] Singh A.S., Mulder C., Twisk J.W., van Mechelen W., Chinapaw M.J. (2008). Tracking of childhood overweight into adulthood: A systematic review of the literature. Obes. Rev..

[4] Lanting C.I., de Vroome E.M., Elias S.G., van den Brandt P.A., van Leeuwen F.E., Kampman E., Kiemeney L.A., Peeters P.H., de Vries E., Bausch-Goldbohm R.A. (2014). Contribution of lifestyle factors to cancer: Secondary analysis of Dutch data over 2010 and a projection for 2020. Ned. Tijdschr. Geneeskd..

[5] Singer M.R., Moore L.L., Garrahie E.J., Ellison R.C. (1995). The tracking of nutrient intake in young children: The Framingham children’s study. Am. J. Public Health.

[6] Statistics Netherlands Minder Kinderen naar Kinderdagverblijven (Less Children in Day Care), 2014. http://www.cbs.nl/nl-NL/menu/themas/dossiers/jongeren/publicaties/artikelen/archief/2014/2014-4024-wm.htm.

[7] Gubbels J.S., Raaijmakers L.G.M., Gerards S.M.P.L., Kremers S.P.J. (2014). Dietary intake by Dutch 1- to 3-year-old children at childcare and at home. Nutrients.

[8] Gubbels J.S., Gerards S.M., Kremers S.P. (2015). Use of food practices by childcare staff and the association with dietary intake of children at childcare. Nutrients.

[9] WebArchitecten vof. FoodFigures. http://www.foodfigures.nl/.

[10] SAS (1999–2001.). Version 8.2.

[11] SAS (1999–2001.). Version 9.3.

[12] Kistemaker C., Bouman M. (1999). Voedselconsumptiepeiling Met SAS. de Ontwikkeling van een Geautomatiseerd Systeem voor de Verwerking van Voedingsenquetes. Deel 2: Beschrijving Functioneel en Systeem Ontwerp (Vertrouwelijk). Rapportnummer V98.799.

[13] NEVO-Online Versie 2013/4.0. http://nevo-online.rivm.nl/.

[14] Dekkers A.L.M., Verkaik-Kloosterman J., Rossum C.T.M., Ocke M.C. (2014). SPADE, a new statistical program to estimate habitual dietary intake from multiple food sources and dietary supplements. J. Nutr..

[15] EFSA Panel on Dietetic Products, Nutrition and Allergies (NDA) (2010). Scientific opinion on principles for deriving and applying dietary reference values. EFSA J..

[16] Health Council of The Netherlands (2001). Dietary Reference Intakes: Energy, Proteins, Fats and Digestible Carbohydrates.

[17] Health Council of The Netherlands (2006). Guideline for Dietary Fibre Intake.

[18] Voedingscentrum (Netherlands Nutrition Centre) Aanbevelingen voor Vitamine, Mineralen en Spoorelementen: Factsheet (Recommendations for Vitamins, Minerals, and Trace Elements), 2014. http://www.voedingscentrum.nl/nl/pers/factsheets.aspx.

[19] EFSA NDA Panel (EFSA Panel on Dietetic Products, Nutrition and Allergies) (2013). Scientific opinion on nutrient requirements and dietary intakes of infants and young children in the European Union. EFSA J..

[20] Gezond eten met de Schijf van Vijf. http://www.voedingscentrum.nl/nl/schijf-van-vijf/schijf.aspx.

[21] Voedingscentrum (Netherlands Nutrition Centre) (2011). Richtlijnen Voedselkeuze, 1 Maart 2011, Update 12 April 2011 (Guidelines for Food Choice, 12 April 2011).

[22] EFSA NDA Panel (EFSA Panel on Dietetic Products, Nutrition and Allergies) (2015). Scientific opinion on dietary reference values for iron. EFSA J..

[23] Encyclopedie. http://www.voedingscentrum.nl/encyclopedie/zout.aspx.

[24] Ocké M.C., van Rossum C.T.M., Fransen H.P., Buurma E.M., de Boer E.J., Brants H.A.M., Niekerk E.M., van der Laan J.D., Drijvers J.J.M.M., Ghameshlou Z. (2008). Dutch National Food Consumption Survey—Young Children 2005/2006.

[25] Portegijs W., Cloïn M., Merens A. (2014). Krimp in de Kinderopvang.

[26] Lozoff B., Jimenez E., Smith J.B. (2006). Double burden of iron deficiency in infancy and low socioeconomic status: A longitudinal analysis of cognitive test scores to age 19 years. Arch. Pediatr. Adolesc. Med..

[27] Qubty W., Renaud D.L. (2014). Cognitive impairment associated with low ferritin responsive to iron supplementation. Pediatr. Neurol..

[28] Trumbo P., Yates A.A., Schlicker S., Poos M. (2001). Dietary reference intakes: Vitamin A, vitamin K, arsenic, boron, chromium, copper, iodine, iron, manganese, molybdenum, nickel, silicon, vanadium, and zinc. J. Am. Diet. Assoc..

[29] Uijterschout L., Vloemans J., Vos R., Teunisse P.P., Hudig C., Bubbers S., Verbruggen S., Veldhorst M., de Leeuw T., van Goudoever J.B. (2014). Prevalence and risk factors of iron deficiency in healthy young children in the southwestern Netherlands. J. Pediatr. Gastroenterol. Nutr..

[30] Weber M., Grote V., Closa-Monasterolo R., Escribano J., Langehendries J., Sain E., Giovannini M., Verduci E., Gruzfeld S., Socha P. (2014). Lower protein content in infant formula reduces BMI and obesity risk at school age: Follow-up of a randomized trial. Am. J. Clin. Nutr..

[31] Hebestreit A., Börnhorst C., Barba G., Siani A., Huybrechts I., Tognon G., Eiben G., Moreno L.A., Fernández Alvira J.M., Loit H.M. (2014). Associations between energy intake, daily food intake and energy density of foods and BMI *z*-score in 2–9-year-old european children. Eur. J. Nutr..

[32] De Ruyter J.C., Olthof M.R., Seidell J.C., Katan M.B. (2012). A trial of sugar-free or sugar-sweetened beverages and body weight in children. N. Engl. J. Med..

